# CRISPR-Cas9 directed knock-out of a constitutively expressed gene using lance array nanoinjection

**DOI:** 10.1186/s40064-016-3037-0

**Published:** 2016-09-09

**Authors:** John W. Sessions, Craig S. Skousen, Kevin D. Price, Brad W. Hanks, Sandra Hope, Jonathan K. Alder, Brian D. Jensen

**Affiliations:** 1 Department of Mechanical Engineering, Brigham Young University, Provo, UT USA; 2 Department of Microbiology and Molecular Biology, Brigham Young University, Provo, UT USA; 3 Department of Physiology and Developmental Biology, Brigham Young University, Provo, UT USA

**Keywords:** Lance array nanoinjection, CRISPR-Cas9, Gene knock-out, Serial injection, Current control, Non-viral transfection

## Abstract

**Background:**

CRISPR-Cas9 genome editing and labeling has emerged as an important tool in biologic research, particularly in regards to potential transgenic and gene therapy applications. Delivery of CRISPR-Cas9 plasmids to target cells is typically done by non-viral methods (chemical, physical, and/or electrical), which are limited by low transfection efficiencies or with viral vectors, which are limited by safety and restricted volume size. In this work, a non-viral transfection technology, named lance array nanoinjection (LAN), utilizes a microfabricated silicon chip to physically and electrically deliver genetic material to large numbers of target cells. To demonstrate its utility, we used the CRISPR-Cas9 system to edit the genome of isogenic cells. Two variables related to the LAN process were tested which include the magnitude of current used during plasmid attraction to the silicon lance array (1.5, 4.5, or 6.0 mA) and the number of times cells were injected (one or three times).

**Results:**

Results indicate that most successful genome editing occurred after injecting three times at a current control setting of 4.5 mA, reaching a median level of 93.77 % modification. Furthermore, we found that genome editing using LAN follows a non-linear injection-dose response, meaning samples injected three times had modification rates as high as nearly 12 times analogously treated single injected samples.

**Conclusions:**

These findings demonstrate the LAN’s ability to deliver genetic material to cells and indicate that successful alteration of the genome is influenced by a serial injection method as well as the electrical current settings.

## Background

The creativity and scale with which researchers are utilizing clustered regularly interspaced short palindromic repeat (CRISPR) sequences and Cas9 (CRISPR-associated) proteins for genomic editing has led to an explosion of possibilities in both transgenic research and gene therapy applications (Feng et al. 2015; Horii et al. 2013; Li et al. 2015; Mou et al. 2015; Nicholson et al. 2015; Petersen and Niemann 2015; Seruggia and Montoliu 2014). Three major elements fueling this movement include the target versatility and ease with which researchers can generate CRISPR-Cas plasmids (Ran et al. 2013), the ability to modify multiple genomic locations in a single step (Ousterout et al. 2015; Wang et al. 2013), and the ability to do so at rates difficult to obtain using other editing methods such as transcription activator-like effector nucleases (TALEN) (Ding et al. 2013; Pennisi 2013).

Despite the great potential CRISPR/Cas9 plasmids offer, there are limitations that make delivering molecular loads to target cells challenging for widespread application. Commonly used viruses, such as adenoviruses, adeno-associated viruses, and lentiviruses, are known for having high transfection rates (Deyle and Russell 2009; Matrai et al. 2010). However, adenoviruses cause excessive immune reactions (Ritter et al. 2002), adeno-associated viruses can cause insertional mutagenesis (Deyle and Russell 2009), and lentiviruses can cause both immune reactions and insertional mutagenesis (Follenzi et al. 2007; Hacein-Bey-Abina et al. 2003; Matrai et al. 2010; VandenDriessche et al. 2002). While CRISPR-Cas9 provides an elegant method to by-pass many of the concerns related to insertional mutagenesis (Zhou et al. 2014), viruses are still constrained by payload capacity (Gardlik et al. 2005), which could limit utility.

In an effort to address concerns raised with viral transduction, researchers have put emphasis on developing chemical, physical, and/or electrical transfection technologies aimed at producing a robust delivery method in terms of effective delivery and expression without compromising cell viability (Itaka and Kataoka 2009; Park et al. 2010; van Gaal et al. 2011). Unfortunately, the trade-off for using non-viral approaches has resulted in lower transfection rates (Mellott et al. 2013) and additional challenges, such as genetic load transfer and preservation across both the cell and nuclear membranes (Ferrari et al. 2002; Ferrer-Miralles et al. 2008; Kodama et al. 2006; Lungwitz et al. 2005; Pouton and Seymour 2001). Commonly used chemical methods, such as cationic lipids and polymers, can be effective in transfection (Baoum et al. 2010, 2012; Baoum and Berkland 2011) (although not as effective as viral modalities (Godbey and Mikos 2001; Godbey et al. 1999; Green et al. 2008; Jo and Tabata 2008; Merdan et al. 2002; Middaugh and Ramsey 2007; Midoux et al. 2009; Park et al. 2006)), but are also potentially toxic to cells because of dosage requirements, usually require optimization experimentation for each cell type, and do not work in all cell lines (Mellott et al. 2013; Wiethoff and Middaugh 2003). Physical methods like microinjection and electrical methods like electroporation can be effective (Mehier-Humbert and Guy 2005), but often are traumatic to the target cells, reducing cell viability (Mellott et al. 2013).

**Fig. 1:**
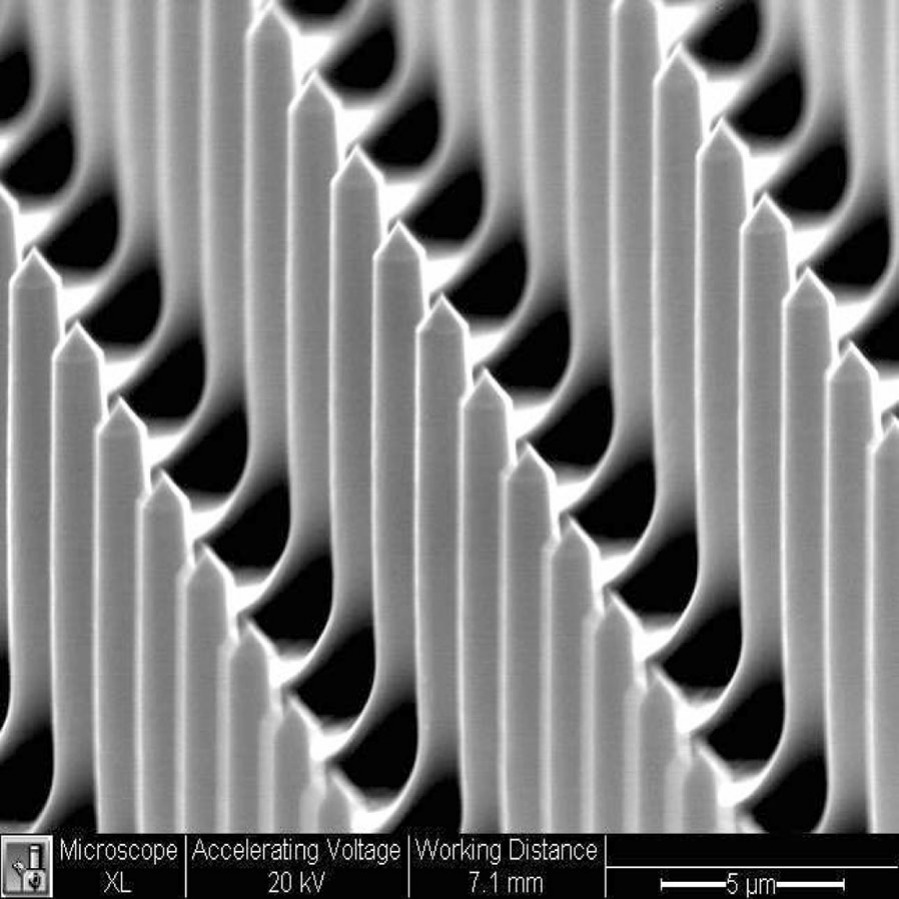
Isometric projection of silicon etched lance array taken by scanning electron microscope. Lances measure 8–10 µm in length and 1–2.5 µm in diameter. Spacing of lances from center-to-center measure 10 µm in both planar directions in a grid of 2000 by 2000 lances per chip

Recently, a new non-viral transfection technology, known as lance array nanoinjection (LAN) was introduced which was designed with many of these challenges in mind. LAN uses a microfabricated silicon etched array of lances to physically penetrate hundreds of thousands of cells simultaneously and electrically deliver attached molecular loads (Lindstrom et al. 2014; Sessions et al. 2014; Teichert et al. 2013) (see Fig. 1). Built upon a first generation technology used to create transgenic mice (Aten et al. 2011, 2012; Wilson et al. 2013), LAN interacts directly with the molecular load via electrical interactions, thereby eliminating viral-induced immune responses and carrier-vehicle cytotoxicity issues. Furthermore, LAN creates transient pores between 1 and 2.5 µm in diameter (making it possible to deliver large loads) and it has cell survival rates of 78–91 % post-injection (Lindstrom et al. 2014).

In this report, we extend previous work by combining LAN with CRISPR-Cas9 technology. To do this, a CRISPR-Cas9 plasmid was designed to knock-out (KO) constitutive green fluorescent protein (GFP) expression in HeLa cells via Non-Homologous End-Joining (NHEJ) repair. Two major variables explored in this work included: the current-control setting used during the initial attraction of DNA to the silicon lances prior to cell membrane penetration (1.5, 4.5, and 6.0 mA) and the number of times samples were injected (one time, x1; three times, x3). We report that cells injected x3 had a significantly higher number of cells with GFP knocked-out when compared to samples x1 injected samples and that the injection-dose response was non-linear. Also, it was observed that an intermediate current control setting (4.5 mA) used during the LAN process produced the greatest percentage of living, GFP negative HeLa cells.

## Methods

### Lance array nanoinjection of cells

For injections, cells were seeded on coverslips and mounted on a platform in a well filled with media. DNA was added to the well with the lance array positioned above the cells. Upon DNA attraction to the lance tips using an electrical charge, the lance array was injected into the cells on the coverslip. Once the lance tips penetrated the cell membrane, the charge on the lance tips reversed to release DNA according to the experimental group defined below. After removing the lance array, the coverslip with injected cells was then removed from the injection well and placed in a culture dish until cells were harvested and flow cytometry readings were taken to determine the ability of the injected CRISPR DNA to knock out expression of the gene of interest.

### GFP+/FRT HeLa cell line

We generated an isogenic cell line containing a single copy of EGFP (enhanced green fluorescent protein) by cloning the code sequence of EGFP into pCDNA5/FRT and introducing this plasmid into HeLa/FRT cells in the presence of Flip recombinase (Flp-In System, Life Technologies, Carlsbad, CA). Hygromycin selected HeLa cells expressed 99 % GFP and were grown in Dulbeccos Modified Eagles Medium (DMEM, Life Technologies, Carlsbad, CA) with 10 % Fetal Bovine Serum (FBS, Denville Scientific, Holliston, MA) and streptomyocin/penicillin (Gibco, Waltham, MA) and incubated at 37 °C and 5 % carbon dioxide.

### CRISPR plasmid

In order to facilitate GFP knock-out in the GFP+/FRT HeLa cell line, a CRISPR-Cas9 plasmid was constructed using sgRNA primers directed towards the N-terminus of EGFP, which would disrupt GFP production via NHEJ repair inaccuracies (Mali et al. 2013; Ran et al. 2013).

The CRISPR-Cas9 GFP knock-out plasmid was created by preparing and ligating sgRNA GFP oligos to a pSpCas9(BB)-2A-Puro (PX459) plasmid, a gift from Feng Zhang (Addgene plasmid #48139) (Ran et al. 2013). The top and bottom oligos were prepared to be inserted into the pSpCas9(BB)-2A-Puro plasmid using protocol previously described in the literature (Ran et al. 2013). Top10 cells were then transformed with plasmids using a standard transformation protocol. DNA was amplified and extracted from the Top10 cells following the protocols of Qiagen Maxi and Mega prep kits (Qiagen, Valencia, CA).

### Injection set-up

**Fig. 2:**
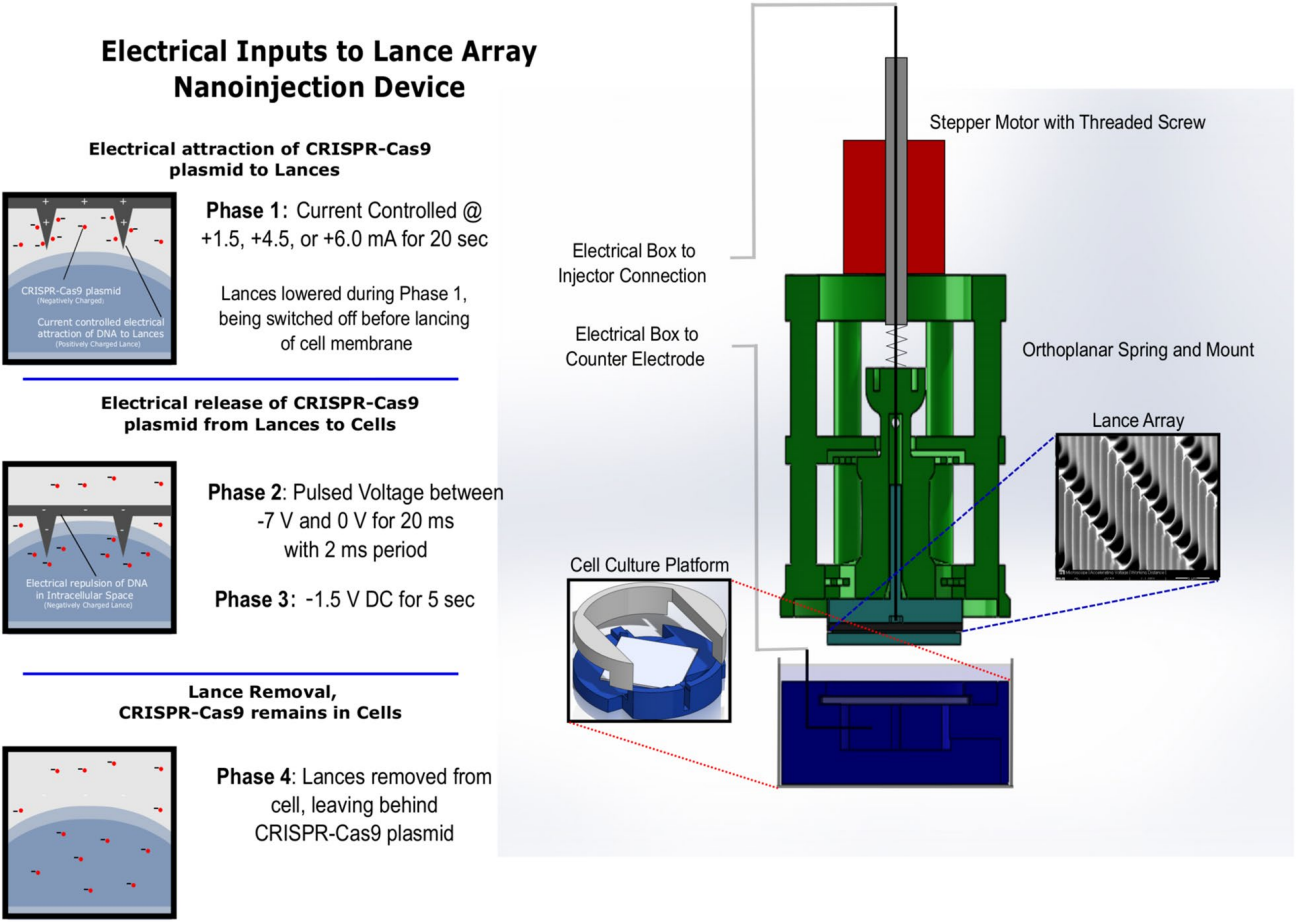
Diagram of the LAN set-up. On the *left*, the four phases of the LAN process in terms of electrical parameters and physical events are described. Illustration of the connection of the stepper motor to the orthoplanar spring is shown on the *right*. With the lance array pointed downward, during the injection process, the lances are inserted into cultured cells secured on the cell culture platform

The LAN device used for injections is comprised of five major components which include: silicon-etched lance array, orthoplanar spring and mount, cell culture platform, stepper motor and threaded screw, and electrical switch box. Figure 2 illustrates the interactions of these components and provides a context to the LAN process.

### Silicon-etched lance, orthoplanar spring and mount

The LAN device contains a microfabricated silicon wafer with etched lances (Fig. 1) attached to an orthoplanar spring which has the stepper motor mounted on top (Fig. 2). The lance array serves to physically penetrate cell membranes and also to electrically interact with DNA. The orthoplanar spring has an attachment on its bottom surface for the silicon lance chip to be inserted, thereby providing vertical motion required for injection as well as the electrical connections. Construction of both the lance array and the orthoplanar spring are discussed in prior literature (Teichert et al. 2013; Teichert and Jensen 2013).

### Cell culture platform

The cell culture platform consists of three individual pieces: two PLA 3D printed platform pieces (MakerBot Replicator 2, MakerBot, Brooklyn, NY) (snap-fit together) and the glass slide (contains adhered cells). The system is designed such that the glass slide can be assembled into the cell culture platform during injection, which helps with alignment of the orthoplanar spring/lance array attachment. Following injection, the glass slide can be easily removed from the assembly for incubation.

### Stepper motor and threaded screw

The stepper motor is shown in Fig. 2 as a component attached to the top surface of the orthoplanar spring and serves as an actuator of the spring in orchestration with the electrical input signals delivered to the silicon lances. The stepper motor is controlled by an Arduino Uno (Small Projects, Somerville, MA) and has been calibrated to vertically operate the threaded screw insert at 160 µm/s.

### Electrical box

The electrical box is designed to take electrical input signals from three different power sources (Keithley 2400, Cleveland, OH) and to output them to the two electrical leads; one lead passing through the upper portions of the injection device to supply charge to the lance array, and another lead passing through the cell culture platform to act as a counter-electrode beneath the cell culture. Figure 2 describes the electrical conditions supplied by the electrical box during the four phases of the injection process. The timing of the electrical signal delivery to the nanoinjection device is controlled by an Arduino Uno.

### Testing preparation

GFP Positive/FRT HeLa cells were prepared approximately 24 h in advance on 18 mm by 18 mm glass slides contained in six well plates. The n-number for an experimental group refers to the number of individual glass slides subjected to the treatment and analysis as described below. Cells were incubated during this period at 37C, 5 % carbon dioxide, and supplied with 2 mL of DMEM with 10 % FBS and streptomyocin/penicillin. Cells cultures on the glass slides were approximately 70 % confluent.

Following this 24 h incubation, the HeLa cells were snapped into the 3D-printed cell culture platforms. Following transfer to the platforms, cells were supplied with 2 mL of fresh DMEM and 4 µL of 25 mM chloroquine and then incubated for an additional 15 min. After being pre-treated with chloroquine, DMEM was removed and 2 mL of phosphate buffered solution (PBS, Gibco, Waltham, MA) was added. Incubation for an addition 15 min followed.

Immediately prior to injection, treatment samples were supplied with the CRISPR-Cas9 plasmid at a concentration of 750 ng of DNA/mL of PBS injection solution.

### Post testing flow cytometry preparation

Following injection, all samples had their glass slides removed from 3D-printed cell culture platforms and placed into six-well plates with 2 mL of DMEM. All treatment samples were then incubated for a period of 7 days to allow the existing GFP to be lost from cells.

After 7 days of incubation, all samples were trypsinized with 0.5 mL of 5× trypsin (Sigma Aldrich, St. Louis, MO) per well and incubated for 5 min. Trypsin was then deactivated with 1.5 mL of DMEM per well and then transferred to FACS tubes for centrifugation for 10 min at 2000 rpm. Following centrifugation, samples’ supernatant were removed and cells were re-suspended in 0.5 mL of PBS with 80 µL of propidium iodide (PI, 500 µg/mL; Sigma Aldrich, St. Louis, MO). The PI served as a viability stain for selecting living cells from dead cells in post flow cytometry analysis.

### Flow cytometry

All samples were quantified using flow cytometry (Attune Acoustic Focusing Flow Cytometer, Life Technologies, Carlsbad, CA). Prior to flow, appropriate single color samples were generated for GFP and PI in order to compensate for signal cross-over. Each sample was then run and had approximately 20,000 events counted and characterized.

**Fig. 3:**
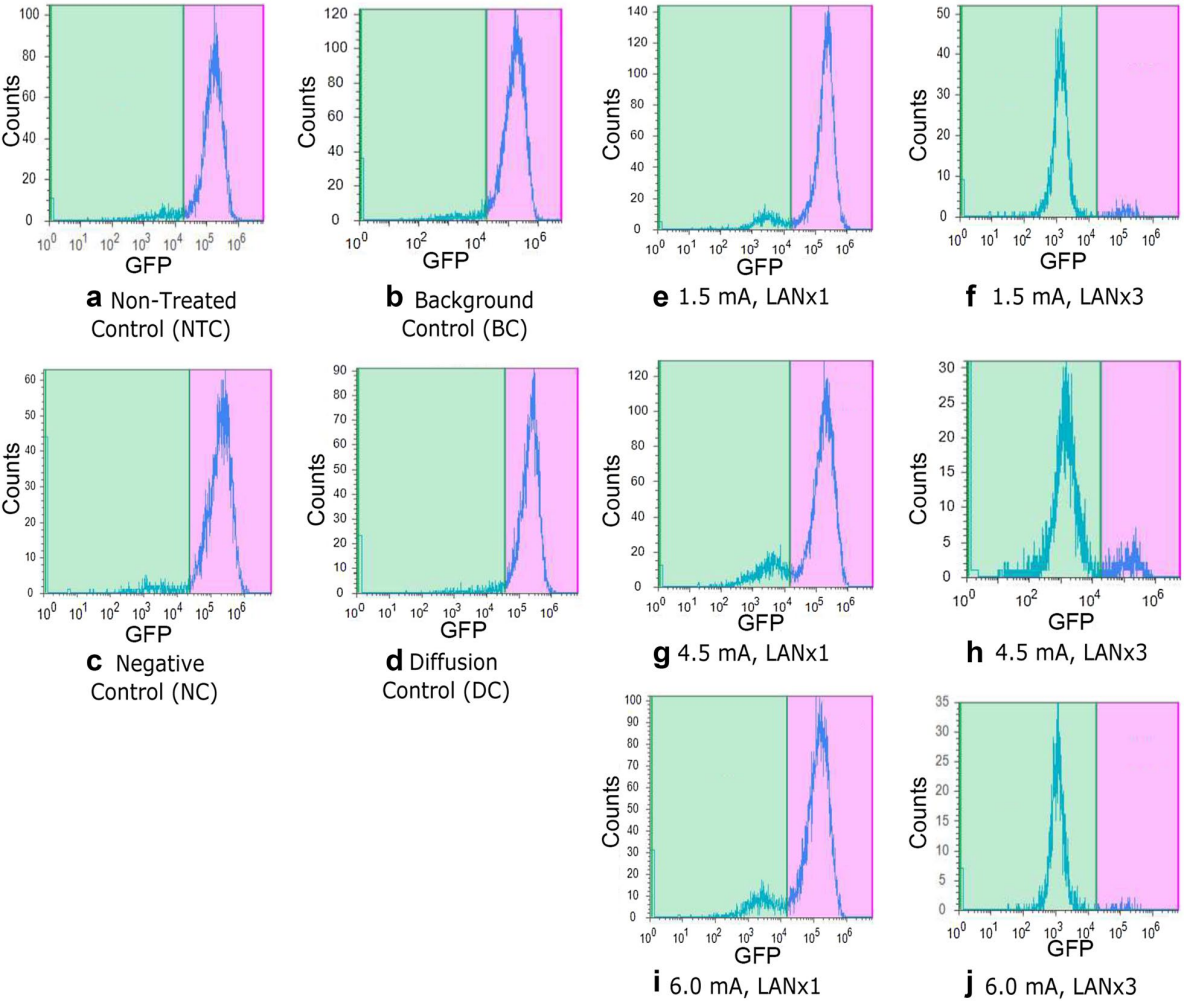
Flow cytometry analysis to determine gene expression changes. Histograms of raw data were divided to determine the number of GFP negative (*left side*) and GFP positive (*right side*) cells on the plot for each sample. The number of cells expressing GFP did not decrease in control samples (**a**–**d**). Side-by-side, experimental samples exhibited a significant increase in GFP negative cells when injected three times (LANx3) compared to cells injected once (LANx1) in 1.5 mA injections (**e**, **f**), 4.5 mA injections (**g**, **h**), and 6.0 mA injections (**i**, **j**). Compare to data in Table 1 and Fig. 4

Using Attune’s post-processing software, samples were gated based on PI signals for living vs dead cells. Using only the living cell populations, samples were then gated based on the GFP signal in order to characterize the efficacy of the CRISPR-Cas9/LAN knock-out of GFP. Refer to Fig. 3 for example flow cytometry results based on the gating procedure described.

### Statistical analysis

Data gathered from flow cytometry was analyzed statistically in JMP (SAS, Cary, NC) using an ANOVA test (F-ratio = 48.0318) and Student *t* tests (α = 0.05). The efficiency statistic reported in the “Results” section is based on number of living GFP Negative cells in each sample. Of note, based on the hypotheses that the samples injected three times will be greater than or equal to the samples injected once, *t* tests involving the comparison of the controls to treatment groups or treatment groups against one another, a one-tailed *p* value is reported.

Samples were allowed to incubate for 7 days post-injection to ensure GFP degradation. Due to this long period of time for cell division, cells were passed in order to prevent crowding on the cover slips; therefore, relative viability rates from the injection are not available. Expression data is reported as a percentage of the living cells in the final sample harvested at 7 days. For context of viability rates following LAN, other works have reported viability rates post-injection between approximately 75–90 % (Sessions et al. 2014).

## Results

Ten experimental groups were created in order to characterize the effects that the number of injection events and the current control settings used during LAN would have on GFP knock-out in the GFP Positive/FRT HeLa cells. Four of these groups were controls, which consisted of: Non-Treated Controls (NTC)—received no injection, no applied current/voltage, and no DNA; Background Controls for nanoinjection (BC)—received DNA, but no applied electrical protocols or physical injection; Negative Controls for electrical exposure (NC)—received physical injection, but with no applied electrical protocols or DNA; and lastly Diffusion Controls (DC)—received physical injection and DNA, but no applied electrical protocol.

Treated samples make up the other six experimental groups. Single Injection (x1) samples were physically injected only one time under the following conditions:

- Phase 1: 20 s application of 1.5, 4.5, or 6.0 mA across lance array (performed with lances external to cell) for the purpose of DNA collection on the lance.
- Phase 2: Lance array inserted into cell culture and pulsed with 10 square-wave pulses of amplitude between 0 and −7 V for 20 ms.
- Phase 3: Directly following pulsing, a 5 s period of −1.5 V DC is applied
- Phase 4: Lance array is removed from the cells.

Multiple Injection (x3) samples were Injected under the same conditions as described for Single Injections (x1) with the exception that following the first injection, cell cultures were placed back into the incubator for 1 h before injecting again. This process was repeated for a total of 3 injection events into the same cell culture.

For convenience with treatment sample nomenclature, specific experimental groups will be referred to by the current control setting used during Phase 1 and the number of times the sample was injected. For instance, 1.5 mA, x1 means that the sample received 1.5 mA during Phase 1 and the samples were nanoinjected only once.

### Multiple LAN injections are more effective than one injection

The construction of the HeLa cell line was noted earlier, and consisted of a pFRT/laczeo HeLa cell line (Flp-In System, Life Technologies) which was purchased and transfected with a pcDNA5/FRT expression vector containing both the gene of interest and means to facilitate its production, which include: a green fluorescent protein (GFP) gene, a CMV promoter, and a FRT site. The FRT site is situated upstream of the compliment ATG sequence for the hygromycin gene found in the Flp-In host HeLa cell line. Co-transfection with the pOG44 vector expresses the flip recombinase machinery that allows the pcDNA5/FRT to flip into the pFRT/laczeo HeLa cell line, thereby making the hygromycin gene in the HeLa cells functionally complete. Once both transfection events were accomplished, HeLa cells that successfully had flipped-in the GFP gene at the FRT site could be selected by hygromycin.

The construction of the CRISPR-Cas9 plasmid was noted earlier, and consisted of a pSpCas9(BB)-2A-Puro (PX459) plasmid, a gift from Feng Zhang (Addgene plasmid #48139) (Ran et al. 2013) that was modified with oligos that code for sgRNA that directs Cas9 to the FRT site to disrupt GFP production via NHEJ. If the CRISPR-Cas9 GFP knock-out plasmid is successful in disrupting the GFP gene at the targeted FRT site, the HeLa cells will become GFP negative because these HeLa cells only have a single GFP gene.

Table 1 summarizes the flow cytometry results of the respective experimental groups demonstrating the disruption of the GFP gene. Two key findings were noted in regards to the number of injections and transfection rates. First, the transfection rates for the x3 injected samples are significantly higher than the x1 injected samples, with the 4.5 mA, x3 treatment samples achieving the highest observed mean knock-out with a median GFP knock-out efficiency of 93.77 % (see Table 1). Figure 3 provides an example of the flow cytometry results from each experimental group used to obtain the data in Table 1.

**Table 1:** LAN Statistical summary of the sample types and associated GFP KO rates

Sample type	Sample size (n)	Mean GFP KO percent (%)	Median GFP KO percent (%)
NTC	21	5.27	5.37
NC	26	3.92	3.62
BC	18	5.96	5.37
DC	23	4.04	3.82
1.5 mA, x1	16	6.92	6.11
4.5 mA, x1	8	21.63	17.37
6.0 mA, x1	16	22.65	8.45
1.5 mA, x3	20	66.79	72.78
4.5 mA, x3	27	79.56	93.77
6.0 mA, x3	20	70.01	70.32

Data collected from flow cytometry and analyzed in JMP represents mean and median GFP KO rates for the respective sample types. Percentage of cells successfully transfected is calculated as the number of living and GFP negative cells divided by the number of living cells in each sample

Data collected from flow cytometry and analyzed in JMP represents mean and median GFP KO rates for the respective sample types. The percentage of cells successfully transfected is calculated as the number of living and GFP negative cells divided by the number of living cells in each sample.

Second, Table 2 demonstrates that a large difference was observed between mean values for all experimental groups when examining the effect of the number of injections as determined by flow (Fig. 3). The largest difference was observed in the 1.5 mA samples, exhibiting a change of 59.87 % when comparing x1 to x3 injected samples. When viewed in context of Table 1, it is noted that the injection-dose response of all treatment samples is non-linear, meaning the rate of GFP knock-out did not follow a linear scale with the number of times cells were injected. In the case of the 1.5 mA current controlled samples, the single injection mean transfection rate is 6.11 %. On a linear scale, the predicted value for samples injected three times should be roughly 18.33 %. Instead, for 1.5 mA, x3 treatment samples the transfection rate is 72.78 %, nearly 4 times greater than the linear predicted value. Similar but less pronounced observations were made in the case of the 4.5 and 6.0 mA samples, resulting in differences in linear predictions and observed mean transfection rates of 14.67 and 2.06 %, respectively.

**Table 2:** One-sided *T* test results from comparisons of multiple (x3) versus single (x1) injected samples

Multiple injections (1) versus single injections (2)	*P* value	Difference in mean GFP KO (1–2) (%)
1.5 mA, x3 versus 1.5 mA, x1	<0.0001	59.87
4.5 mA, x3 versus 4.5 mA, x1	<0.0001	57.93
6.0 mA, x3 versus 6.0 mA, x1	<0.0001	47.36

Represented data was initially screened in JMP using ANOVA test to determine presence of statistically significance relationships followed by one-sided *t* test (α = 0.05) evaluation of specific comparisons. Default minimum *p* value reported is 0.0001. All represented relationships are statistically different

Represented data was initially screened in JMP using ANOVA test to determine presence of statistically significance relationships followed by one-sided *t* test (α = 0.05) evaluation of specific comparisons using the full data set. All represented relationships are statistically different.

### Mid-range current control yielded the highest observed knock-out

Results in Table 3 show the statistical comparisons between sample types, grouped according to the number of times the samples were injected. While it is of note that there were no intra-group comparisons that were statistically significant in regards to current control effects on GFP knock-out (with the exception of 1.5 mA, x1 vs 6.0 mA, x1 injections), the relative position of the median values (Table 1) for both the single and multiple injection groups appears to favor the 4.5 mA treated samples. The data suggests that using an intermediate current control setting during injection may improve transfection efficiency.

**Table 3:** One-sided *T* test results from intra-group comparisons (by number of times injected)

Single (x1) injected comparisons	*P* value	Multiple (x3) injected comparisons	*P* value
1.5 mA, x1 versus 4.5 mA, x1	0.0684	1.5 mA, x3 versus 4.5 mA, x3	0.0985
1.5 mA, x1 versus 6.0 mA, x1	0.0093	1.5 mA, x3 versus 6.0 mA, x3	0.3454
4.5 mA, x1 versus 6.0 mA, x1	0.4621	4.5 mA, x3 versus 6.0 mA, x3	0.8895

Represented data was initially screened in JMP using ANOVA test to determine presence of statistically significance relationships followed by one-sided *t* test (α = 0.05) evaluation of specific comparisons. Default minimum *p* value reported is 0.0001

Only one statistically significant relationship was identified between the 1.5 mA, x1 and 6.0 mA, x1

## Discussion

Much like other transfection methods, the designed intent of LAN is to direct genetic loads into target cells without threatening their survival. Noted earlier, viruses have been a mainstay in transfection protocols because of the higher transfection rates that can be achieved relative to non-viral modalities (Mellott et al. 2013). LAN is a non-viral method designed to address this short-coming by generically delivering any electrically charged molecular load by electrostatic attraction and release into the intracellular space of target cells via small micron-sized lance structures.

This process of electrical interaction with genetic material and physical penetration of the cell membrane was originally created for mouse embryonic transgenic research using a microelectromechanical system (MEMS)-based single silicon lance (Aten et al. 2008). Using this device, it was shown that nanoinjection had comparable transfection rates to microinjection but increased embryo survival rates (Aten et al. 2012). This delivery system was particularly useful because injections merely needed to be cytoplasmic due to the localized electroporative effect of the lance on the pronuclei—an event termed intracellular electroporetic nanoinjection (IEN) (Wilson et al. 2013).

Later, nanoinjection was extended to somatic cell targets by utilizing an array of silicon etched lances, a design used in this work. Electrostatic principles used to initially determine DNA behavior with the single lance injector (Aten et al. 2011; David et al. 2010) have been also applied in LAN, both structurally (Teichert et al. 2013; Teichert and Jensen 2013) and procedurally (Lindstrom et al. 2014; Sessions et al. 2014).

Initial experimentation with LAN was designed to show the effectiveness with which small molecules (such as propidium iodide, PI) could be delivered to cultured cells and what impact injection would have on survival. As mentioned in the materials and methods section, relative viability rates from this study are not available because cells incubated 7 days post-injection divide to the extent that the original cell death would not be observable. To give a context of cell survival for this study, previous results demonstrated a voltage–dependent relationship for injection success, while maintaining viability rates between 78 and 91 % (Lindstrom et al. 2014). Similar results were obtained using LAN in experimentation designed to assess the effects of different saline solution types used during injection (Sessions et al. 2014).

This current work marks the first LAN proof-of-concept results regarding the use of CRIPSR-Cas9 plasmids to knock-out gene function. Table 1 indicates that the maximum median GFP KO efficiencies of 70.32–93.77 % can be achieved using LAN after injecting HeLa cells three times.

Contextually, these LAN efficiencies are encouraging because high-throughput screening of the human genome using CRISPR-Cas9 plasmids designed to knock-out (KO) gene function have proven to be critical to understanding gene function (Zhou et al. 2014). Maggio et al. (2014) recently demonstrated the KO behavior of CRISPR-Cas9 using adenoviral vector delivery of gRNA and Cas9 in two separate vectors. Designed to target the AAVS1 “safe harbor” locus in a panel of human cells types which include: cervix carcinoma HeLa cells, osteosarcoma U2OS cells, hMSCs, and myoblasts, this team showed relative gene KO when increasing amounts of the two vectors were applied. In the case of HeLa cell experimentation, Maggio et al. achieved maximum gene KO of 31 % when 100 TU/cell of both vectors, an efficiency rate less than a third of the maximum efficiency reported here using LAN in the same cell type. While our study did not provide a direct comparison between the two methods of transfection, the data is compelling towards further development of the LAN as a new technology in delivering genetic material into cells.

**Fig. 4:**
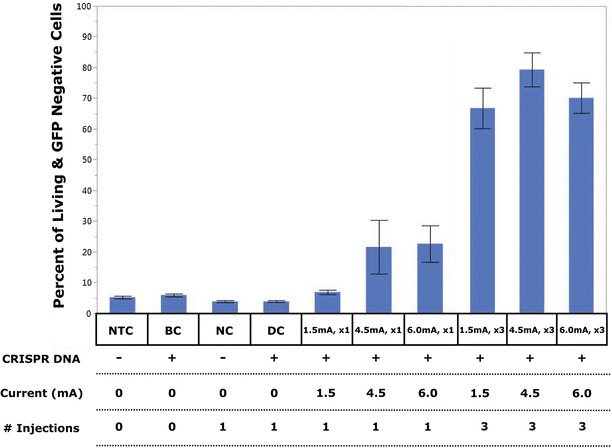
Lance array nanoinjection delivers CRISPR DNA for gene expression knock out. The percentage of GFP negative cells within the viable population is plotted for each experimental group (mean and SEM). Controls include non-treated control (NTC), DNA background control (BC), negative electrical exposure control (NC), and DNA diffusion control (DC). Fully injected samples include cells injected once or three times with 1.5, 4.5, or 6.0 mA as the current on the LAN for release of DNA into the cells. Statistically significant relationships are noted in Tables 2 and 3

In addition to exhibiting high knock-out efficiency rates, this work also shows in Fig. 4 the non-linear difference between the x1 and x3 injected samples, a behavior not previously noted in LAN. For example, 1.5 mA, x1 samples had a median KO rate of 6.11 %, while 1.5 mA, x3 samples had a median KO rate of 72.78 %, a rate nearly 12 times higher. While the magnitudes are not as high for the other sample comparisons, the non-linear trend is still present.

One possible explanation for this behavior is related to cellular response to cell membrane defects, such as defects created by lance induced pores. When a surface defect occurs in a cell membrane, the cell responds by attempting to mobilize and remodel structural elements such as actinomyosin, microtubules, and the cell membrane by contracting around the wound and allowing repair machinery to close membrane gaps (Abreu-Blanco et al. 2011a, b). In LAN samples that experience multiple injections, one possible explanation for increased GFP KO is that repair mechanisms may be delayed because of prior insults still being repaired. If true, saturation of repair mechanisms would permit longer periods of time for CRISPR-Cas9 plasmid movement into the cell following multiple injection treatments, and thereby allowing greater GFP KO. While this idea potentially explains why greater plasmid delivery may be possible, it does assume that diffusion is a major factor in plasmid motion across a cell membrane following LAN, a behavior that is not supported by the diffusion controls (DC) used in this study.

Another explanation for the increased GFP KO with samples injected x3 is related to how quickly the target cells remove the delivered plasmid. Prior to injection, samples are pre-treated with chloroquine, an agent designed to inhibit lysosomal action, to increase the half-life of the plasmid in the cytoplasm of target cells. It is possible that initial dosing of plasmids into target cells during the first injection event is enough to saturate functional lysosomes such that when additional plasmids are delivered in subsequent injection events, higher levels of functional CRISPR-Cas9 plasmids are available to disrupt GFP gene function. Again, this idea has not been defined in prior work and requires further investigation.

Another behavior noted in regards to non-linear KO rates deals with the current control exposure during Phase 1. Noted in Table 1 is the fact that 1.5 mA samples experience the greatest increase in GFP KO from x1 injection treatment to x3 injection treatment (mean difference of 59.87 %). It has been noted in previous work that lower applied electrical conditions during LAN contribute to higher cell viability rates (Sessions et al. 2014). It is believed that the because the 1.5 mA, x3 treatment samples received a reduced electrical exposure during injection, that more successfully transfected cells survived to be GFP negative than the 4.5 and 6.0 mA treated samples. If that were the case, samples exposed to 1.5 mA would more likely survive the injection process and thereby potentially increase the transfection rate.

Intertwined in the discussion of the non-linear behavior of the GFP KO rates when comparing x1 injections to x3 injections is the fact that mid-range current controlled samples had the best median GFP KO. It is observed in Table 2 that the 1.5 mA samples experience the greatest change in GFP KO from x1 to x3 injected samples (a difference of 59.87 %), while overall the 4.5 mA samples experience the greatest magnitude in GFP KO, reaching a median value of 93.77 % for the x3 injected treatment group. A possible explanation for this behavior is that 4.5 mA protocols offers the best balance between effective electrical attraction/release of the DNA during the LAN process, while being a mild stressor in terms of cell viability. Even though the 1.5 mA protocol is milder in terms of cellular stress, a feature seen in electroporation studies to improve cell viability (Canatella et al. 2001), perhaps the 4.5 mA protocol is better at balancing the cellular stress with effective attraction and release of the DNA, a parameter shown to increase DNA motion when done at higher magnitudes in processes like electrophoresis (David et al. 2010, 2011, 2012).

Having demonstrated the ability to effectively KO gene function using CRISPR-Cas9 plasmids, future work regarding LAN may aim to either optimize this reported process or explore other genomic mechanisms that CRISPR-Cas9 can perform, such as transcriptional activation/repression or gene insertion (Cheng et al. 2013; Gilbert et al. 2013; Kimura et al. 2014, 2015; Maeder et al. 2013; Qi et al. 2013), in terms of other cell types, such as primary cell lines or stem cells. Primary cell line targets are of interest because of the potential therapeutic options it creates in regards to gene medicine and gene therapy applications, such as enhancing chronic wound healing (Badillo et al. 2007; Branski et al. 2009; Eming et al. 1995; Eriksson et al. 1998; Galeano et al. 2003). Stem cells are also of interest because of similar gene therapy potentials as well as applications related to transgenic animal generation. Specifically, a common method for creating chimeric transgenic animals involves genetic modification of stem cells prior to introduction to the blastocyst (Guo et al. 2014; Murayama et al. 2015; Ohtsuka et al. 2012; Polejaeva and Mitalipov 2013). The methods used to genetically modify these stem cells (i.e. electroporation, liposomal reagents) are characteristically threatening to cell survivability and/or have lower transfection rates (Hu et al. 2013; Huang et al. 2008; Mellott et al. 2013). As discussed, LAN could provide a viable alternative in this area given the fact that genetic modifications and cell survival can occur at high rates, and therefore requires further investigation.
